# Increased frequency of proangiogenic tunica intima endothelial kinase 2 (Tie2) expressing monocytes in individuals with type 2 diabetes mellitus

**DOI:** 10.1186/s12933-022-01497-6

**Published:** 2022-05-12

**Authors:** M. Reijrink, J. van Ark, C. P. H. Lexis, L. M. Visser, M. E. Lodewijk, I. C. C. van der Horst, C. J. Zeebregts, H. van Goor, S. C. A. de Jager, G. Pasterkamp, B. H. R. Wolffenbuttel, J. L. Hillebrands

**Affiliations:** 1 Department of Pathology & Medical Biology - Pathology, University of Groningen, University Medical Center Groningen, Hanzeplein 1, PO Box 30.001, Groningen, The Netherlands; 2 Department of Internal Medicine – Division of Vascular Medicine, University of Groningen, University Medical Center Groningen, Groningen, The Netherlands; 3 Department of Cardiology, University of Groningen, University Medical Center Groningen, Groningen, The Netherlands; 4 Department of Intensive Care Medicine, Cardiovascular Research Institute Maastricht (CARIM), Maastricht University, Maastricht, The Netherlands; 5 Department of Intensive Care Medicine, Maastricht University Medical Center, Maastricht, The Netherlands; 6 Department of Surgery – Division of Vascular Surgery, University of Groningen, University Medical Center Groningen, Groningen, The Netherlands; 7 Laboratory of Experimental Cardiology, University Medical Center Utrecht, University of Utrecht, Utrecht, The Netherlands; 8 Center Diagnostic Laboratory, Division Laboratories and Pharmacy, University Medical Center Utrecht, Utrecht University, Utrecht, The Netherlands; 9 Department of Endocrinology, University of Groningen, University Medical Center Groningen, Groningen, The Netherlands

**Keywords:** Angiogenesis, Atherosclerosis, Macrovascular disease, Monocytes, Monocyte heterogeneity, Tie2, Type 2 diabetes mellitus

## Abstract

**Background:**

Individuals with type 2 diabetes mellitus (T2DM) have an increased risk for developing macrovascular disease (MVD) manifested by atherosclerosis. Phenotypically and functionally different monocyte subsets (classical; CD14^++^CD16^−^, non-classical; CD14^+^CD16^++^, and intermediate; CD14^++^CD16^+^) including pro-angiogenic monocytes expressing Tie2 (TEMs) can be identified. Here we investigated monocyte heterogeneity and its association with T2DM and MVD.

**Methods:**

Individuals with (N = 51) and without (N = 56) T2DM were recruited and allocated to "non-MVD" or "with MVD" (i.e., peripheral or coronary artery disease) subgroups. Blood monocyte subsets were quantified based on CD14, CD16 and Tie2 expression levels. Plasma levels of Tie2-ligands angiopoietin-1 and angiopoietin-2 were determined using ELISA. Carotid endarterectomy samples from individuals with (N = 24) and without (N = 22) T2DM were stained for intraplaque CD68^+^ macrophages (inflammation) and CD34^+^ (angiogenesis), as plaque vulnerability markers.

**Results:**

Monocyte counts were similar between individuals with T2DM and healthy controls (non-diabetic, non-MVD). Non-classical monocytes were reduced (p < 0.05) in T2DM, whereas the percentage of TEMs within the intermediate subset was increased (p < 0.05). T2DM was associated with increased angiopoietin-1 (p < 0.05) and angiopoietin-2 (p = 0.0001) levels. Angiopoietin-2 levels were higher in T2DM individuals with MVD compared with non-MVD (p < 0.01). Endarterectomized plaques showed no differences in macrophage influx and microvessel number between individuals with and without T2DM.

**Conclusions:**

Monocyte subset distribution is altered in T2DM with reduced non-classical monocytes and increased TEM percentage in the intermediate monocyte subset. Increased angiopoietin-2 levels together with increased frequency of TEMs might promote plaque vulnerability in T2DM which could however not be confirmed at tissue level in advanced atherosclerotic lesions.

## Background

Individuals with type 2 diabetes mellitus (T2DM) are at increased cardiovascular risk compared to individuals without T2DM as a result of accelerated development of macrovascular disease (MVD), including coronary artery disease (CAD) and peripheral artery disease (PAD) [1, 2]. The formation of microvessels (i.e., angiogenesis) in atherosclerotic plaques contributes to the development of MVD and plaque vulnerability, with local hypoxia and inflammation including infiltrating monocytes as driving forces [3]. Neoangiogenesis (from the adventitia towards the plaque) contributes to atherosclerotic plaque progression and intraplaque haemorrhage by microvessel incompetence which might also result in plaque rupture [4, 5]. Once extravasated into tissues, monocytes differentiate into inflammation-associated M1 macrophages or anti-inflammatory, angiogenesis-associated M2 macrophages [6], although this dichotomy is most likely oversimplified and other macrophage subtypes may exist. The pathogenetic effects of infiltrating macrophages include loading with lipids resulting in transformation into foam cells, which might be enhanced by hyperglycemia and hypercholesterolemia [7]. Foam cells trapped in a plaque may die and form a necretoic core. Circulating monocytes, as precursors of macrophages, form a heterogeneous subpopulation of circulating myeloid cells which play a critical role in the pathogenesis of atherosclerosis and thereby contribute to the development of cardiovascular disease (CVD) [8–11]. Monocytes can be divided into 3 subpopulations based on the differential expression of CD14 (LPS co-receptor) and CD16 (FcγIIIR). The main population (70–90%) of monocytes is negative for CD16 but is characterized by high CD14 expression (CD14^++^CD16^−^); this main monocyte population is referred to as "classical monocytes". CD14^++^ monocytes which express relatively low levels of CD16 (CD14^++^CD16^+^) are called "intermediate monocytes" whereas a third population with low CD14 but high CD16 expression (CD14^+^CD16^++^) are so-called "non-classical monocytes" [12, 13]. These monocyte subtypes have different functional properties, express different levels of chemokine receptors, have different gene expression profiles, and secrete different cytokines upon stimulation [14, 15]. Due to their functional diversity the different subtypes are likely to contribute differently to the atherogenic process [16]. An association has been observed between monocyte subset frequency and cardiovascular outcome in several diseases. This association is complex, and it is not fully elucidated whether the role of different subsets is protective or detrimental [17–21]. A recent systematic review and meta-analysis concluded that an increased number of intermediate and non-classical monocytes is most likely present in individuals with cardiometabolic disorders such as T2DM and individuals with CVD, based on an increased production of pro-inflammatory factors [22].

Previously a monocyte subpopulation was identified based on the expression of angiopoietin receptor tunica intima endothelial kinase 2 (Tie2, TEK, CD202B) [23]. Tie2 is a cellular membrane receptor tyrosine kinase and has molecular effects such as cell–cell and cell–matrix interactions, cell proliferation, migration and survival [24]. Next to that, Tie2 affects endothelial cells and is an important factor in vasculogenesis and in exhibition of defects in angiogenesis and vascular remodelling as well as in vessel integrity [25]. Tie2 positive cells are able to differentiate into mesenchymal lineages such as adipogenic, chrondogenic and, osteogenic lineages [26]. Tie2^+^ monocytes can be detected in all CD14/CD16 monocyte subsets, but are enriched in the CD14^++^CD16^+^ intermediate population [27]. These so-called Tie2 expressing monocytes (TEMs) have been shown to infiltrate tumours and exert proangiogenic functions [28]. Therefore, TEMs contribute to tumour angiogenesis by secreting proangiogenic growth factors that promote matrix remodelling, endothelial sprouting, and capillary formation. This angiogenic function was shown to be enhanced by stimulation with Tie2 ligand angiopoietin-2 (Ang2), both in vitro and in vivo [29–31]. Angiopoietin-1 (Ang1) is expressed by pericytes, fibroblasts, stromal cells and tumour cells and has anti-inflammatory effects, generates neovascularization and contributes to vascular homeostasis and protection [32]. Ang2 is primarily expressed by endothelial cells, which is induced by hypoxia and shear stress. In contrast to Ang1, Ang2 stimulates production of inflammatory mediators and this ligand might even counteract the anti-inflammatory effects of Ang1, leading to upregulation of adhesion molecules and induction of leukocyte adhesion [32]. In light of the association of monocyte heterogeneity with inflammatory diseases and atherosclerosis, alterations in monocyte subsets could be involved in the augmented development of MVD in individuals with T2DM. However, the association of skewed monocyte heterogeneity and the presence of MVD in T2DM has not been investigated. While TEMs in individuals with T2DM were recently reported [33, 34], to our best knowledge no data on TEMs in T2DM in relation to inflammation and angiogenesis have been published so far. Based on these observations we tested the overall hypothesis that T2DM is associated with altered monocyte subset distribution in favour of proinflammatory and proangiogenic monocyte subsets, especially in individuals suffering from MVD. First, we tested whether CD14^++^CD16^+^ intermediate monocytes, associated with increased cardiovascular risk [17, 18, 22, 35], were increased in individuals with T2DM, particularly in individuals with MVD. Secondly, we tested the hypothesis that the frequency of proangiogenic Tie2^+^ monocytes is increased in individuals with MVD and T2DM and whether this is associated with circulating levels of Tie2-ligands Ang1 and Ang2. Finally, endarterectomized human carotid plaques obtained from individuals with and without T2DM were analysed for plaque vulnerability characterized by CD68^+^ macrophage infiltration and angiogenesis.

## Methods

### Aim, design and setting of the study

The primary aim of this study was to investigate monocyte heterogeneity in peripheral blood in six different groups with individuals with and without T2DM with and without MVD (i.e. coronary artery disease [CAD] and peripheral artery disease [PAD]). The subdivision in PAD and CAD was made since underlying pathophysiologic processes driving development of these macrovascular complications might differ. Secondly, we aimed to study the association of TEMs and Tie-2 ligands Ang1 and Ang2 with T2DM and MVD. Finally, plaque vulnerability characteristics were investigated in human atherosclerotic plaques from individuals with and without T2DM. This study is performed as part of a single centre, case–control study [36].

### Participant characteristics

In this study, we included a subset of individuals from a cohort on which we reported previously [36]. Characteristics are shown in Table 1. Since the TEM analyses were added to the protocol during the study, these analyses were performed on a smaller subset. No additional in- or exclusion criteria were used for selection of these individuals. Comparison of the total cohort and the TEM subcohort revealed no significant differences (Table 2). Participants were allocated into one of the following 6 groups: T2DM without MVD (N = 15); T2DM with CAD (N = 15); T2DM with PAD (N = 21); non-diabetes without MVD (i.e., healthy control individuals, N = 19); non-diabetes with CAD (N = 16); non-diabetes with PAD (N = 21). Diagnosis of T2DM was based on diagnostic criteria defined by the WHO [37]. CAD was diagnosed based on prior myocardial infarction (> 6 months) or evidence of significant coronary artery stenosis during angiography. PAD was defined as a history of claudication or rest pain and assessed with ultrasonography of bilateral peripheral arterial foot pulses. Individuals with clinical evidence of both CAD and PAD were excluded from the study. In addition, conventional cardiovascular risk factor information of all participants, including smoking, hypertension, and dyslipidaemia were collected.

**Table 1:** Characteristics of study participants

	Flow cytometry analysis (cohort 1)						*p-value*	Carotid endarterectomy (cohort 2)		
	Type 2 diabetes mellitus (T2DM)			Non-diabetes						
	Without MVD (N = 15)	With CAD (N = 15)	With PAD (N = 21)	Healthy controls (N = 19)	With CAD (N = 16)	With PAD (N = 21)		T2DM (N = 24)	Non-diabetes (N = 22)	*p-value* *Cohort 1 vs cohort 2*
Demographics										
Age	58 ± 2.7	66 ± 1.7^a^	67.1 ± 1.6^b^	54.6 ± 1.0	57.1 ± 2.4	59.2 ± 1.6	< 0.001	70 ± 7.2	69 ± 9.7	< 0.001
Male (%)	6 (40)	7 (47)	11 (52)	11 (58)	11 (69)	15 (71)	ns	11 (46)	15 (68)	ns
Body mass index (kg/m^2^)	32.9 ± 1.6	31.2 ± 1.6	31.6 ± 2.4	25.2 ± 0.7^c^	27.0 ± 1.0	23.2 ± 0.7^c^	< 0.001	26.9 ± 3.6	25.8 ± 3.0	ns
Diabetes duration (years)	15.5 ± 1.0	15.1 ± 2.0	12.0 ± 1.8	–	–	–	ns	6.9 ± 5.4 (N = 15)	–	< 0.01
Smoking (%)	5 (33)	2 (13)	5 (24)	3 (16)^h^	9 (56)	14 (67)^h^	< 0.01	8 (33)	6 (27) (N = 23)	ns
Laboratory										
Glucose (mmol/L)	6.4 ± 0.4	8.4 ± 0.8	9.0 ± 1.0^d^	5.5 ± 0.2	–	5.7 ± 0.4	< 0.01	7.2 ± 1.9 (N = 19)	6.6 ± 1.6 (N = 15)	ns
HbA_1c_ (%)	8.0 ± 1.0^e^	7.7 ± 0.3^e^	7.4 ± 0.3^e^	5.7 ± 0.1	5.7 ± 0.1	6.0 ± 0.2	< 0.001	NA	NA	–
HbA_1c_ (mmol/mol)	64.0 ± 5.0^e^	60.5 ± 3.5^e^	57.8 ± 3.4^e^	38.9 ± 0.7	38.6 ± 1.2	42.1 ± 1.8	< 0.001	NA	NA	–
Cholesterol (mmol/L)	4.2 ± 0.2	4.4 ± 0.3	4.1 ± 0.2	5.6 ± 0.2^f^	4.4 ± 0.3	4.7 ± 0.3	< 0.01	4.7 ± 0.6 (N = 13)	4.3 ± 0.9 (N = 15)	ns
Triglycerides (mmol/L)	1.7 ± 0.2	2.5 ± 0.6	1.7 ± 0.1	1.6 ± 0.2	1.8 ± 0.3	1.9 ± 0.3	ns	1.5 ± 0.5 (N = 13)	1.3 ± 0.7 (N = 15)	< 0.05
HDL (mmol/L)	1.3 ± 0.1	1.3 ± 0.2	1.4 ± 0.2	1.6 ± 0.1	1.2 ± 0.1	1.4 ± 0.1	ns	1.2 ± 0.4 (N = 13)	1.3 ± 0.4 (N = 15)	ns
LDL (mmol/L)	2.3 ± 0.1	2.5 ± 0.3	2.2 ± 0.2	3.4 ± 0.2^g^	2.7 ± 0.2	2.7 ± 0.3	< 0.01	2.7 ± 0.7 (N = 14)	2.4 ± 0.8 (N = 13)	ns
Creatinine (μmol/L)	67 ± 4.2	76.4 ± 5.4	72.6 ± 5.2	77.7 ± 3.7	79.7 ± 3.4	75.9 ± 5.0	ns	77.9 ± 17.9	95.6 ± 34.8	< 0.05
Glomerular filtration rate (mL/min/1.73 m^2^)	98.1 ± 7.2	82.2 ± 3.9	118.5 ± 32.2	84.1 ± 4.4	88.4 ± 5.7	94.5 ± 5.9	ns	83.5 ± 25.1	73.3 ± 21.3	ns

CAD = coronary artery disease, HDL = High-Density Lipoprotein, LDL = Low-Density Lipoprotein, MVD = macrovascular disease, NA = not available, ns = not significant, PAD = peripheral artery disease

Statistically significant with ANOVA:

^a^ vs. non-diabetes with CAD, control

^b^ vs. diabetes non-MVD, non-diabetes with CAD, non-diabetes with PAD, Control

^c^ vs. diabetes non-MVD, diabetes with CAD, diabetes with PAD

^d^ vs. control, non-diabetes with PAD

^e^ All diabetes groups vs. non-diabetes groups

^f^ vs. diabetes non-MVD, diabetes with CAD, diabetes with PAD, non-diabetes with CAD

^g^ vs. diabetes non-MVD, diabetes with PAD

^h^ Statistically significant with χ2 test

**Table 2:** Comparison of the total cohort and the TEM subcohort

	Total cohort	TEM subcohort	p-value*
Characteristics			
Age	60 ± 8.9 (n = 109)	61 ± 9.6 (n = 46)	0.533
Male (%)	56 (n = 111)	64 (n = 47)	0.353
Body mass index (kg/m^2^)	27 ± 5.8 (n = 81)	28 ± 4.8 (n = 38)	0.357
Diabetes duration (years)	15 ± 7.0 (n = 50)	15 ± 7.8 (n = 23)	0.999
Smoking (%)	34 (n = 111)	32 (n = 47)	0.778
Glucose (mmol/L)	5.7 ± 2.6 (n = 59)	5.8 ± 1.5 (n = 15)	0.887
HbA_1c_ (%)	6.1 ± 1.3 (n = 95)	6.0 ± 1.5 (n = 36)	0.707
HbA_1c_ (mmol/mol)	43 ± 14 (n = 95)	42 ± 16 (n = 36)	0.700
Cholesterol (mmol/L)	4.4 ± 1.1 (n = 90)	4.5 ± 1.1 (n = 38)	0.639
Triglycerides (mmol/L)	1.6 ± 1.1 (n = 90)	1.5 ± 0.93 (n = 38)	0.624
HDL (mmol/L)	1.3 ± 0.60 (n = 89)	1.2 ± 0.55 (n = 38)	0.380
LDL (mmol/L)	2.4 ± 0.92 (n = 89)	2.7 ± 0.83 (n = 38)	0.086
Creatinine (μmol/L)	75 ± 18 (n = 93)	79 ± 17 (n = 37)	0.248
Glomerular filtration rate (mL/min/1.73 m^2^)	89 ± 66 (n = 92)	91 ± 22 (n = 36)	0.859

*Unpaired T-test (nominal variables) and Chi-square (categorical variables)

For this study, non-fasting venous blood samples were obtained with venepuncture and collected in EDTA Vacutainers. In addition, whole blood differential analysis was performed with a PocH-100i haematology analyser (Sysmex Nederland, Etten-Leur, The Netherlands) and routine laboratory measurements of glucose, HbA1_C_, lipid levels, and serum creatinine were performed. A more detailed description of subject inclusion and exclusion criteria (including advanced microvascular disease, auto-immune diseases, neoplasm, acute or chronic infections, < 6 months surgery or vascular intervention, age > 80 years, < 6 months myocardial infarction, haemodialysis and use of immunosuppressive medication) can be found elsewhere [36].

### Flow cytometry

Fluorescence-activated cell sorting (FACS) staining was performed on whole blood samples. To this end, freshly collected blood was centrifuged (2000 rpm, 10 min, 4 °C), and plasma was aspirated and stored at − 80 °C. Next, samples were washed twice with FACS-buffer (PBS/5% FBS) to remove residual plasma.

After centrifugation, concentrated blood samples were incubated with FcR blocking reagent (Miltenyi Biotech GmbH, Bergisch Gladbach, Germany) to block unspecific Fc receptor-mediated antibody binding. Next, samples were incubated at 4 °C for 30 min with a cocktail of monoclonal antibodies consisting of Peridinin Chlorophyll Protein Complex (PerCP)-conjugated mouse-α-human CD14 (Clone MΦP9, BD Biosciences, Franklin Lakes, NJ, USA), Allophycocyanin (APC)-conjugated mouse-α-human CD16 (Clone 3G8, Biolegend, San Diego, CA, USA) or appropriate fluorochrome-conjugated isotype control antibodies. Because CX3C chemokine receptor 1 (CX3CR1) is a key molecule in monocyte chemotaxis and extravasation in atherosclerosis [38], we additionally quantified the frequency of CX3CR1^+^ cells and CX3CR1 mean fluorescence intensity (MFI) within the total monocyte population and the CD14/CD16 monocyte subpopulations using Phycoerythrin (PE)-conjugated rat-α-human CX3CR1 (Clone 2A9-1, Biolegend). In a subset of samples, we performed flow cytometry for TEMs using PE-conjugated mouse-α-human TIE2 (Clone 83,715, R&D Systems, McKinley Place NE, MN, USA) in a cocktail with the abovementioned antibodies against CD14 and CD16. After incubation with antibodies, erythrocytes were lysed with FACS lysing solution (BD Biosciences), and samples were washed with FACS-buffer and fixed in buffered 2% paraformaldehyde. Samples were stored overnight at 4 °C, and FACS data were acquired within 24 h on a FACSCalibur cytometer (BD Biosciences). For analysis of CD14/CD16 monocyte subsets, 1 × 10^5^ cytometric events were acquired; for quantification of Tie2^+^ monocytes 5 × 10^5^ cytometric events were recorded. FACS data analysis was performed with Flowjo (Tree Star Inc., Ashland, OR, USA). Monocytes were gated based on the forward/side scatter profile. Monocyte subsets (based on the expression of CD14 and CD16) were identified within the monocyte gate. CX3CR1 expression was quantified within the CD14/CD16 monocyte subsets. For quantifying Tie2 expression within CD14/CD16 monocyte subsets, fluorescence minus one (FMO, Tie2 antibody replaced with isotype control) samples served as a control for gating.

### Ang1 and Ang2 ELISA

Ang1 and Ang2 are ligands for Tie2 and therefore might influence the function of Tie2^+^ monocytes. Plasma levels of Ang1 and Ang2 were measured in a subgroup of individuals with commercial Enzyme Linked Immunosorbent Assay (ELISA) DuoSet kits (R&D Systems). Frozen plasma samples were thawed overnight on ice at 4 °C and subsequently centrifuged (10,000*g*, 10 min, 4 °C) to remove contaminating platelets. Plasma samples were diluted 30-fold (Ang1) and threefold (Ang2) in reagent diluent. The assay was performed according to the manufacturer's instructions, and the colour reaction was developed with *o*-Phenylenediamine dihydrochloride (OPD, Sigma Aldrich, St. Louis, MO, USA) as substrate. All samples and standards were measured in duplicate, and absorbance values were measured at 492 nm with a VarioSkan Microplate Reader (Thermo Scientific, Landsmeer, The Netherlands).

### Immunohistochemistry for CD68 and CD34

Sections (3 μm thickness) were cut from 46 paraffin-embedded endarterectomized atherosclerotic carotid plaques from individuals with (N = 24) and without (N = 22) T2DM. Samples were selected from the Athero-Express biobank (University Medical Center Utrecht, Utrecht, The Netherlands) [39] and matched the best possible with the characteristics of the cases used for flowcytometric analysis (as shown in Table 1). Sections were stained for CD68 (macrophage infiltration as a marker of inflammation [7]) and CD34 (microvascular endothelial cells, marker of intraplaque angiogenesis [40]). Sections were mounted on glass slides, deparaffinised in xylene, and rehydrated in a graded ethanol series. For CD68 stainings, heat-induced antigen retrieval was performed by incubation overnight in Tris HCl pH9 at 80 °C. After cooling down, endogenous peroxidase activity was blocked in H_**2**_O_**2**_ 0.03% for 30 min, and sections were then incubated with monoclonal mouse anti-human CD68 (#M0876 clone PMG1, DAKO, 1:1000) in 1% human serum/1%BSA/PBS for 60 min at room temperature. Next, 30 min incubation was performed with polyclonal antibody rabbit anti mouse (RAM)-bio (#E0413, DAKO, 1:100) in AB serum and 1%BSA and then with 30 min incubation with streptavidin alkaline phosphatase (AP) (#D0396, DAKO, 1:100) in AB serum and 1%BSA, both at room temperature. AP activity was visualized by incubation for 30 min with Fast Blue BB chromogen solution (10 mg Naphthol AS-MX in 50 mL 0.1 M Tris–HCl, pH 8.2, 4 drops of 10% MgSO_4_ and 50 mg Fast Blue BB and 12 mg Levamisol). For CD34, sections were stained using a Ventana Benchmark automatic stainer. Sections were processed following the standard protocol with primary antibody human CD34 (#790-2927, clone QBEnd/10, Ventana), followed by secondary steps**:** ultraVIEW Universal HRP Multimer (#253-4290, Ventana) and DAB Detection kit (ultraVIEW universal DAB; chromogen, H_2_O_2_, and copper, Ventana). After incubation with the chromogen 3,3’-diaminobenzidine, haematoxylin counterstaining was performed. Sections stained for CD68 were covered with non-aqueous Kaiser's glycerol-gelatin (37 °C) and a coverslip, and sections stained for CD34 sections were dehydrated with ethanol and covered with Tissue-Tek Film (Sakura, CoverSlipper). Sections were digitalized with a Nanozoomer S360 (Hamamatsu, Japan) slide scanner. Digitalized sections were analysed with the ImageScope software package (Aperio v12.4.3.5008, Leica Biosystems Imaging, USA). Atherosclerotic plaque surface area was calculated by manually marking the region of interest, i.e., plaque tissue between the vascular lumen and internal elastic lamina. Numbers of CD34^+^ vessels were counted manually and expressed as number (N) and calculated as N per surface area (N/mm^2^). For CD68 quantification, the standard algorithm Positive Pixel Count 2004-08-11 (version 8.100) was used. The number of strong positive (NSP) pixels was counted as representation of the number of CD68^+^ macrophages and data were expressed as a ratio to surface area (NSP/µm^2^).

Of note, CD68 stainings were performed as double labelling combined with the anti-human Tie2 polyclonal goat antibody (1:20, IgG R&D systems AF313), which was detected using peroxidase-labelled rabbit-anti-goat secondary antibody and visualized with Vector® NovaRED® Substrate kit peroxidase. However, use of the polyclonal antibody resulted in serious nonspecific background staining precluding proper and reliable quantification. It was, therefore, decided only to quantify the amount of CD68 based on the positive Fast Blue BB signal.

### Statistics

Means from normally distributed numeric variables between two groups were compared using an unpaired Student's t-test or Mann–Whitney U-test when not normally distributed. Two-sided Fisher's Exact Tests were performed to compare categorical variables (i.e., gender and smoking status). Multiple testing corrections were used for comparisons between three or more groups ANOVA with Bonferroni. The Pearson's χ^2^ test was used for categorical variables. Differences were considered statistically significant when p-values were < 0.05. All analyses were performed with SPSS (version 20) and GraphPad Prism software (version 9).

## Results

### Patient characteristics

Characteristics of the study populations are shown in Table 1. Individuals with T2DM and CAD were older (66 yrs ± 1.7) than CAD individuals without T2DM (57.1 yrs ± 2.4) and healthy control individuals (54.6 ± 1.0 yrs, p < 0.001). Individuals with T2DM and PAD (67.1 yrs ± 1.6) were older than individuals with T2DM without MVD (58 yrs ± 2.7) and individuals without T2DM (54.6 yrs ± 1.0, p < 0.001). Healthy control individuals and individuals without T2DM with PAD also had a lower BMI than individuals with T2DM (p < 0.001, as shown in Table 1). HbA1_C_ levels were higher in individuals with T2DM than individuals without T2DM (p < 0.001, as shown in Table 1). Healthy control individuals had the highest total cholesterol and LDL cholesterol levels.

### Monocyte subsets

**Fig. 1:**
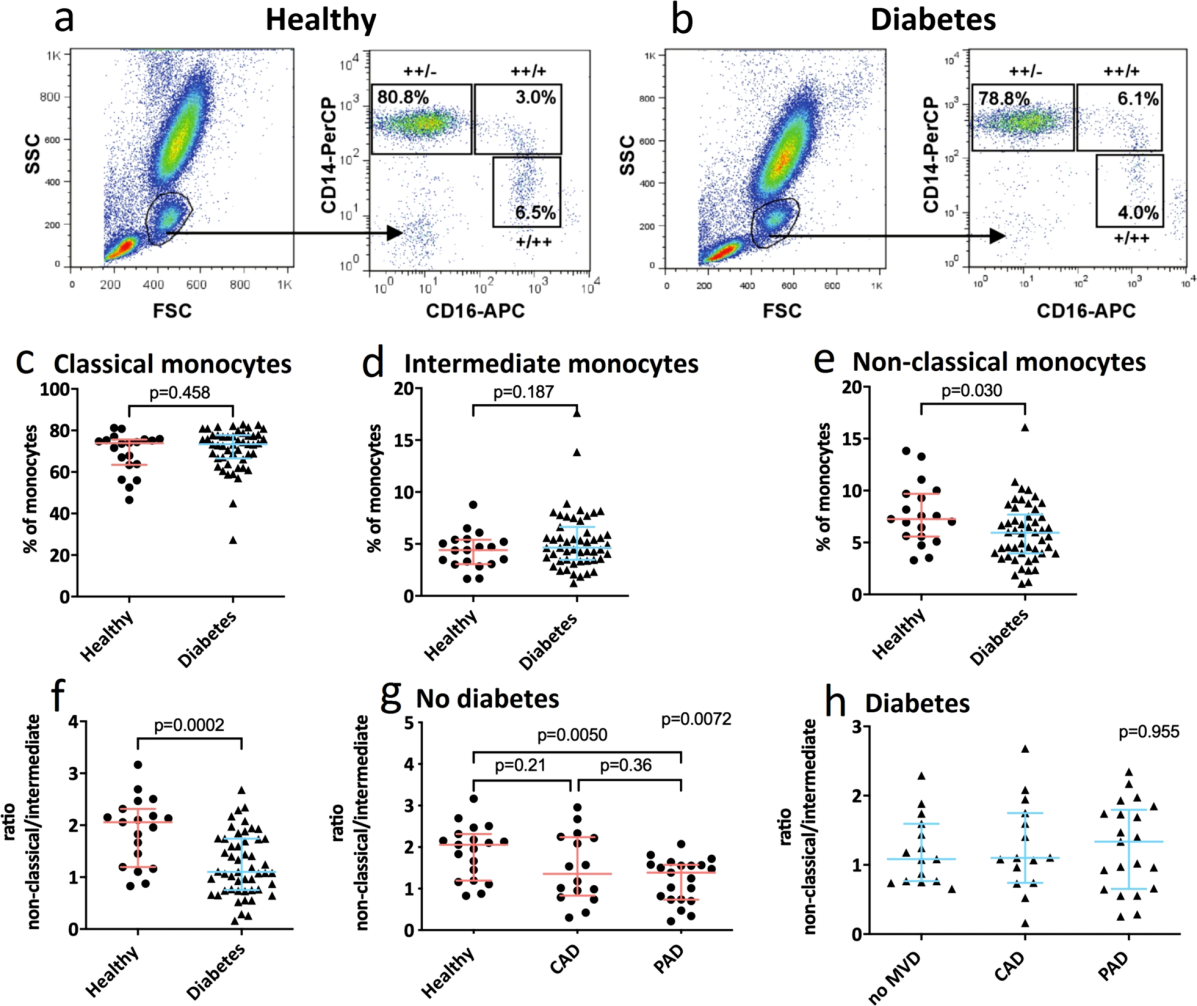
Monocyte subset gating and quantification. Representative fluorescence-activated cell sorting plots and gating strategy for the quantification of monocyte subsets in healthy control individuals (**a**) and individuals with type 2 diabetes mellitus (T2DM) without macrovascular disease (MVD) (**b**). Left panel: Side Scatter (SSC) versus Forward Scatter (FSC) dot plots were used to gate the monocyte population. Right panel: Within the monocyte population, 3 monocyte subsets were gated based on CD14 and CD16 expression. No difference in the percentage of classical monocytes (+±) (**c**) and intermediate monocytes (++/+) between individuals with T2DM and healthy controls was observed (**d**). The percentage of non-classical monocytes (+/++) was 1.3-fold lower in individuals with T2DM compared to healthy controls (**e**). The ratio of non-classical/intermediate monocytes was 1.5-fold lower in individuals with diabetes compared to healthy controls (**f**). In individuals without diabetes, the ratio of non-classical/intermediate monocytes was 1.6-fold lower in individuals with PAD compared to healthy controls (**g**). Within individuals with T2DM, no difference in the ratio of non-classical/intermediate monocytes between individuals with or without MVD was observed (**h**)

Total white blood cell (WBC) counts, differential analysis, and flow cytometry analysis data are presented in Table 3. Individuals with T2DM and PAD had significantly higher counts of total WBCs (in counts × 10^6^ per mL) compared to healthy control individuals (9.1 ± 0.7 vs 5.9 ± 0.4, p < 0.05). This could be attributed to the significantly increased numbers of granulocytes. Moreover, the absolute number of lymphocytes (× 10^6^ per mL) was significantly higher in individuals without T2DM with PAD (2.7 ± 0.2) compared with individuals with (1.8 ± 0.2) and without T2DM (1.8 ± 0.1) with CAD and healthy control individuals (1.9 ± 0.1, p < 0.01). Using flow cytometry, the monocyte population was identified and gated in the forwards/side scatter dot-plot of red blood cell-depleted whole blood samples (Fig. 1). Within this monocyte population, monocyte subsets were gated based on the expression level of CD14 and CD16. Figure 1a and 1b display representative examples of the gating strategy on samples from a healthy control and a patient with T2DM, respectively. The percentage of monocytes within the total WBC pool was significantly lower in individuals with T2DM with PAD (9.1 × 10^6^ per mL ± 0.7) compared with individuals without T2DM with CAD (6.3 ± 0.4, p < 0.05), which might be attributed to the increased number of granulocytes (Table 3). The absolute number of monocytes per mL was significantly higher in individuals without T2DM with PAD compared to healthy controls (690 ± 62 vs 448 ± 25, p < 0.05), while the absolute number of monocytes was similar between individuals with T2DM and healthy controls. Within the monocyte gate 3 monocyte subsets could be identified based on the level of CD14 and CD16 expression as indicated in Fig. 1a and b.

**Table 3:** Whole blood differential and monocyte subset flow cytometry data

	Type 2 diabetes mellitus (T2DM)			Non-diabetes			p-value
	Without MVD (N = 15)	With CAD (N = 15)	With PAD (N = 21)	Healthy controls (N = 19)	With CAD (N = 16)	With PAD (N = 21)	
White blood cell differential (× 10^6^ per mL):							
Total White Blood Cell	8.0 ± 1.0	6.3 ± 0.4	9.1 ± 0.7^a^	5.9 ± 0.4	6.5 ± 0.6	8.2 ± 0.5	p < 0.05
Lymphocytes	2.3 ± 0.3	1.8 ± 0.2	2.3 ± 0.2	1.9 ± 0.1	1.8 ± 0.1	2.7 ± 0.2^b^	p < 0.01
Mononuclear cells	0.7 ± 0.1	0.6 ± 0.04	0.8 ± 0.1	0.6 ± 0.04	0.7 ± 0.1	0.7 ± 0.1	ns
Granulocytes	4.8 ± 0.9	3.9 ± 0.3	6.0 ± 0.6^a^	3.5 ± 0.3	4.0 ± 0.4	4.4 ± 0.4	p < 0.05
Flow cytometry monocytes							
Total monocytes (% of White Blood Cell)	7.0 ± 0.6	8.1 ± 1.0	6.7 ± 0.6^c^	8.1 ± 0.6	9.6 ± 0.4	7.7 ± 0.5	p < 0.05
Total monocytes (per mL)	563 ± 96	504 ± 45	603 ± 60	448 ± 25	618 ± 46	690 ± 62^a^	p < 0.05
Monocyte subset (% of Monocytes)							
CD16^+^ (% of CD14^+^)	10.0 ± 1.0	13.5 ± 1.5	10.4 ± 1.0	12.1 ± 0.9	14.2 ± 1.6	13.2 ± 1.4	ns
CD14^++^CD16^−^ (classical)	72.2 ± 2.7	70.8 ± 1.9	68.8 ± 2.8	68.9 ± 2.3	74.0 ± 2.3	65.9 ± 2.1	ns
CD14^++^CD16^+^ (intermediate)	4.7 ± 0.6	6.4 ± 0.9	5.0 ± 0.6	4.4 ± 0.4	6.8 ± 1.3	6.1 ± 0.7	ns
CD14^+^CD16^++^ (non-classical)	5.2 ± 0.5^d^	7.1 ± 0.9	5.5 ± 0.6^d^	7.7 ± 0.7	7.5 ± 0.8	7.1 ± 0.9	p < 0.01

CAD: coronary artery disease; MVD: macrovascular disease, ns: not significant; PAD: peripheral artery disease

Statistically significant with ANOVA compared to:

^a^Healthy controls

^b^Diabetes with CAD, non-diabetes with CAD and healthy control

^c^Non-diabetes with CAD

^d^Diabetes with CAD, non-diabetes with CAD or PAD, and healthy control

### The frequency of CD14^+^CD16^++^ non-classical monocytes is decreased in individuals with T2DM

The main population consisted of classical monocytes (CD14^++^CD16^−^). Furthermore, intermediate monocytes (CD14^++^CD16^+^) and non-classical monocytes (CD14^+^CD16^++^) could be distinguished. The percentage of total CD16^+^ cells (intermediate and non-classical monocytes) within the total CD14^+^ monocyte pool was not different among all groups.

When comparing healthy control individuals with individuals with T2DM (with and without MVD pooled), no significant differences in the proportion of classical (Fig. 1c) and intermediate monocytes (Fig. 1d) were observed. However, individuals with T2DM were characterized by a significantly (p < 0.05) reduced percentage of non-classical monocytes compared to healthy control individuals (Fig. 1e). This difference could be attributed to primarily the individuals with T2DM without MVD or with PAD (Table 3).

In order to visualize the skewing of CD16^+^ subsets within the CD16^+^ monocyte fraction, the ratio between non-classical and intermediate monocytes was calculated. As shown in Fig. 1f, this ratio was significantly (p < 0.001) decreased in individuals with T2DM compared to healthy control individuals, indicating a relative increase in intermediate monocytes within the CD16^+^ monocyte fraction in these individuals. In individuals without T2DM, this ratio was significantly (p < 0.05) lower in individuals with PAD compared to healthy control individuals (Fig. 1g). In contrast, in individuals with T2DM, no differences in this ratio were observed between individuals with or without MVD (Fig. 1h).

All monocytes showed expression of CX3CR1 to some extent, and therefore no differences were found in the percentage of CX3CR1^+^ monocytes between study groups. CX3CR1 expression levels were significantly different (p < 0.0001) among the monocyte subsets, with classical monocytes having the lowest CX3CR1 MFI (342.0 ± 12.0), intermediate monocytes having intermediate CX3CR1 MFI (839.2 ± 25.6), and non-classical monocytes having the highest CX3CR1 MFI (1359 ± 28.0) (pooled data from individuals with and without T2DM). However, no significant differences in CX3CR1 expression levels were found between individuals with and without T2DM with or without MVD (data not shown). Taken together, these results show that monocyte phenotype is skewed towards a relative increase in intermediate monocytes in individuals with T2DM and individuals with MVD, while CX3CR1 expression was not affected.

### The population of Tie2^+^ intermediate monocytes is increased in individuals with T2DM

**Fig. 2:**
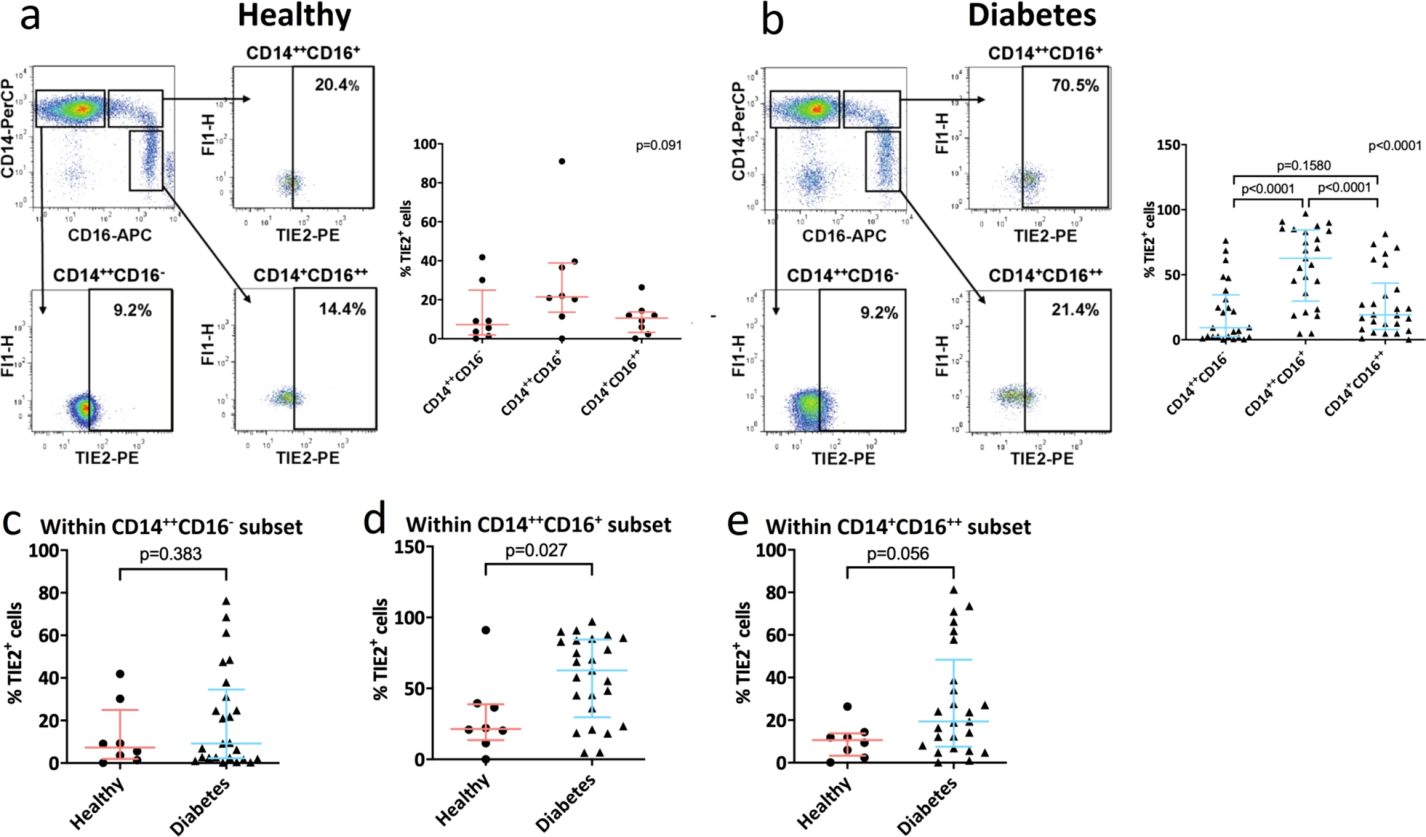
Tie2^+^ monocyte gating and quantification. Representative fluorescence-activated cell sorting plots and gating strategy for the quantification of Tie2^+^ monocytes in healthy control individuals (**a**) and individuals with type 2 diabetes mellitus (T2DM) (**b**). Left 4 panels: Gating of Tie2^+^ monocytes within each monocyte subset, based on isotype controls. Right panel: Graph of Tie2^+^ monocyte frequency within the respective monocyte subset. No differences in Tie2^+^ monocyte frequency within subsets were observed in healthy control individuals. Within individuals with T2DM, the percentage of Tie2^+^ cells was significantly higher within the intermediate monocyte subset compared to the other monocyte populations. The percentage of Tie2^+^ monocytes within the classical monocyte population was similar between healthy individuals and individuals with T2DM (**c**). The percentage of Tie2^+^ cells within in the intermediate monocyte population was 1.9-fold higher in individuals with T2DM compared to healthy control individuals (**d**). The percentage of Tie2^+^ monocytes within in the non-classical monocyte population in individuals with T2DM was increased compared to healthy control individuals, however, without reaching the level of statistical significance (p = 0.056) (**e**)

Within a subgroup of individuals (8 healthy control individuals, 13 individuals with T2DM without MVD, 7 individuals with T2DM with CAD, and 5 individuals with T2DM with PAD), the percentage of Tie2^+^ monocytes within the total monocyte population and the various CD14/CD16 monocyte subsets was quantified. Figure 2 shows representative examples of the gating strategy used to quantify Tie2^+^ monocytes in healthy control individuals (a) and individuals with T2DM (b) within the 3 monocyte subsets.

Within healthy control individuals no differences in the frequency of Tie2^+^ cells among the different monocyte subsets were observed (Fig. 2a, right graph). However, within individuals with T2DM, the frequency of Tie2^+^ monocytes was significantly (p < 0.0001) higher within the intermediate monocyte subset compared with classical and non-classical monocytes (Fig. 2b, right graph).

When comparing the proportion of Tie2^+^ monocytes between healthy control individuals and individuals with T2DM, no significant differences were observed in the percentage of Tie2^+^ monocytes within the total monocyte population (not shown) as well as in the proportion of Tie2^+^ monocytes within the classical monocyte subpopulation (Fig. 2c). However, we observed that the percentage of Tie2^+^ monocytes was significantly increased (p < 0.05) in the intermediate monocyte subset in individuals with T2DM with and without MVD (57.5 ± 5.8% Tie2^+^ cells) compared to healthy controls (30.2 ± 9.8% Tie2^+^ cells, Fig. 2d). In addition, a trend was observed towards increased percentages of Tie2^+^ monocytes in the non-classical monocyte population in individuals with T2DM (p = 0.056, Fig. 2e). Within individuals with T2DM, no differences were detected between individuals with or without MVD with regard to the percentage of Tie2^+^ monocytes within the different CD14/CD16 monocyte subsets (not shown). These results indicate that the percentage of Tie2^+^ monocytes is increased within the intermediate monocyte population in individuals with T2DM regardless of the presence or absence of MVD.

### Circulating Ang1 and Ang2 levels are increased in individuals with T2DM

**Fig. 3:**
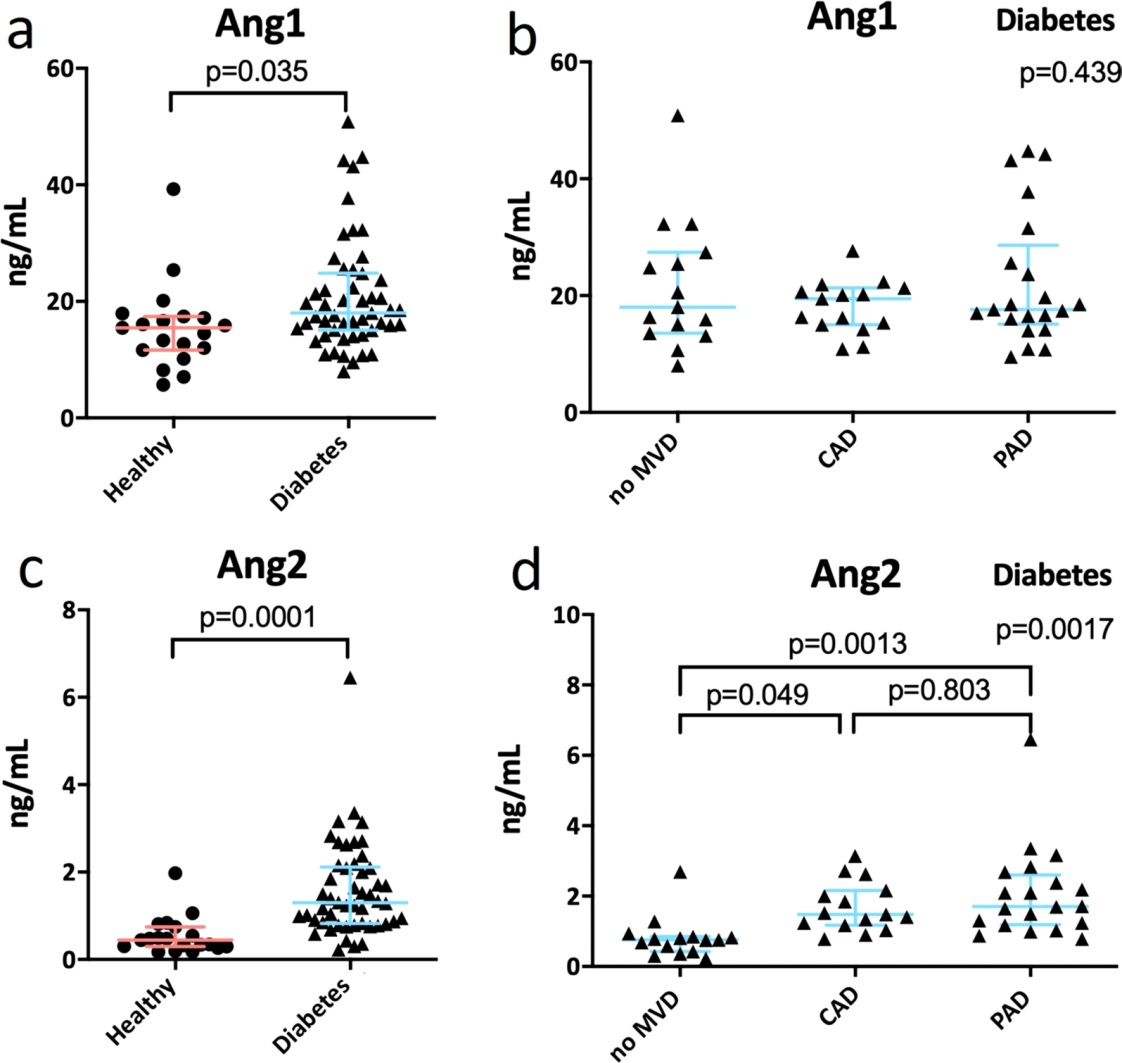
Ang1 and Ang2 plasma concentrations. Ang1 plasma levels were elevated in individuals with T2DM with and without MVD compared to healthy control individuals (1.3-fold increase) (**a**). Within individuals with type 2 diabetes mellitus (T2DM), there was no difference in Ang1 levels between individuals with and without macrovascular disease (MVD) (**b**). Circulating Ang2 levels were 3.2-fold higher in individuals with T2DM compared to healthy controls (**c**). When subdividing individuals with T2DM in individuals with and without MVD, increased Ang2 plasma concentrations were detected in individuals with coronary artery disease (CAD) or peripheral artery disease (PAD) compared with individuals with T2DM without MVD (**d**)

To investigate if Ang1 and Ang2 levels were affected by the presence of diabetes, we determined plasma Ang1 and Ang2 levels in healthy control individuals and individuals with T2DM with and without MVD (Fig. 3). Ang1 levels were higher in individuals with T2DM compared to healthy control individuals (20.9 vs. 15.6 ng/mL respectively, p < 0.05, Fig. 3a). When subdividing individuals with T2DM according to their cardiovascular disease state (non-MVD, CAD and PAD), similar levels of Ang1 among these groups were found (Fig. 3b).

Alike Ang1, also Ang2 plasma concentrations were significantly higher in individuals with T2DM compared to healthy controls (1.6 vs. 0.5 ng/mL respectively, p < 0.001, Fig. 3c). Furthermore, within individuals with T2DM Ang2 levels (in ng/mL) were significantly higher in individuals with CAD (1.69 vs 0.820, p = 0.049) and PAD (2.060 vs 0.820, p = 0.0013) compared to individuals with T2DM without MVD (Fig. 3d). Ang1 and Ang2 levels did not correlate with the frequency of TEMs within the total monocyte pool and the different monocyte subsets. These data show that increased Ang2 levels are associated with vascular disease while circulating Ang1 levels are elevated in diabetes regardless of the presence of MVD.

### Intraplaque CD68^+^ macrophages and CD34^+^ microvessels do not differ between atherosclerotic plaques from diabetic and non-diabetic individuals

Endarterectomised carotid plaques from 24 individuals with T2DM and 22 non-diabetic individuals were analysed for the presence of CD68^+^ macrophages and CD34^+^ vessels. Plaque size did not differ between plaques obtained from individuals with and without T2DM (28 [22–36] mm^2^ and 29 [19–37] mm^2^, respectively (expressed as median [IQR] surface area, p = 0.836)).

**Fig. 4:**
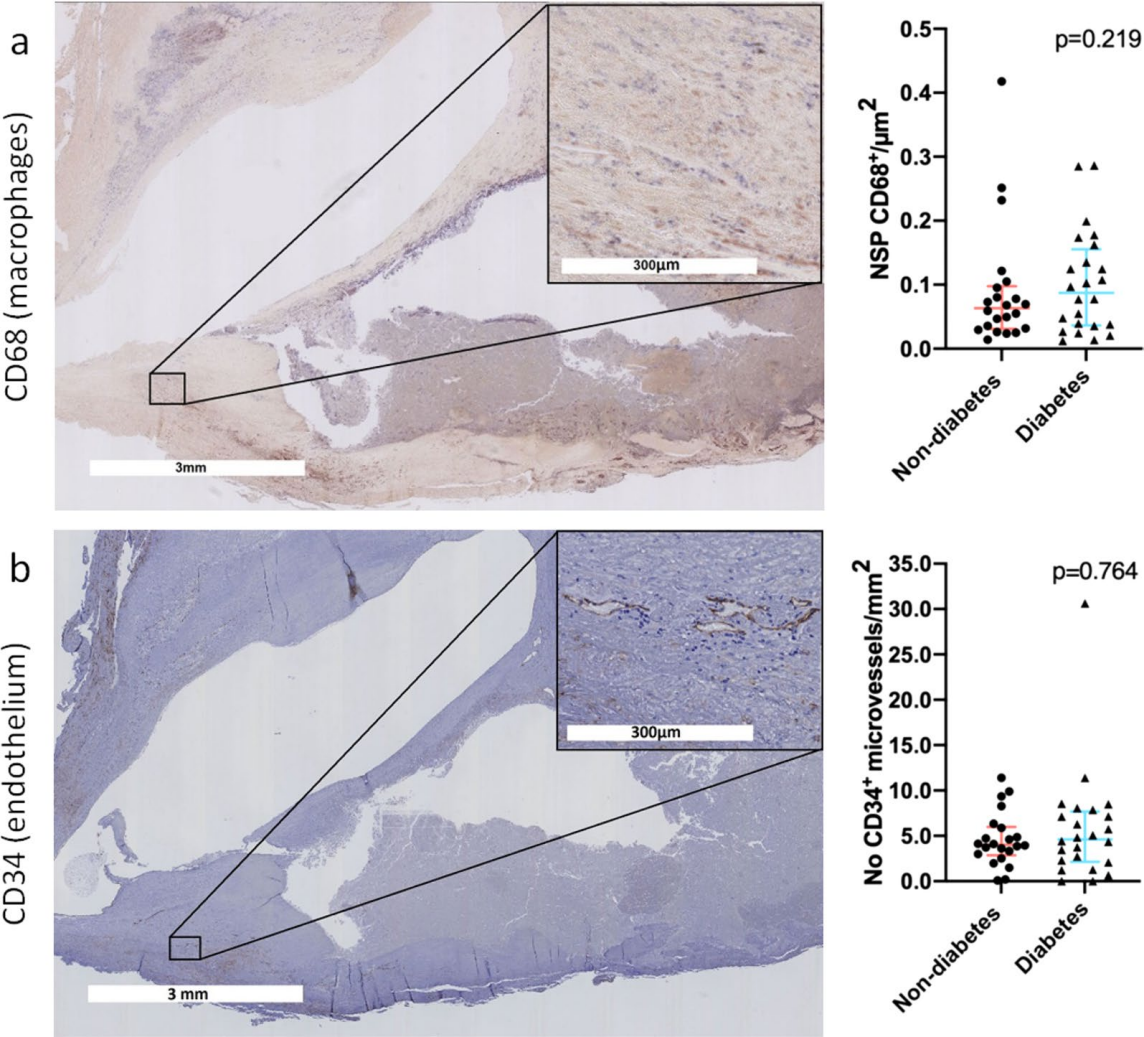
Intraplaque macrophage influx and microvessel formation did not differ between individuals with and without diabetes. The CD68^+^ macrophage number of strong positive pixels (NSP, purple blue coloured) per µm^2^ surface area as a marker of intraplaque inflammation in carotid plaques of individuals without and with type 2 diabetes mellitus (T2DM) (**a**). CD34^+^ microvessel (dark brown coloured) number per mm^2^ surface area as a marker of intraplaque angiogenesis in carotid plaques obtained from individuals without and with T2DM (**b**)

Median [IQR] NSP CD68^+^ macrophages/µm^2^ were 0.087 [0.036–0.16] for plaques from individuals with T2DM vs. 0.063 [0.031–0.098] for plaques from individuals without diabetes (p = 0.219), as shown in Fig. 4a. Absolute numbers of CD34^+^ microvessels did not differ between plaques from individuals with and without T2DM: 127 [80–178] in plaques from individuals with T2DM vs. 109 [56–151] in plaques from individuals without diabetes (p = 0.503, expressed as N median [IQR]). Also, when expressed per surface area, no differences were observed; 4.6 [2.1–7.7] in plaques from individuals with T2DM vs. 4.0 [2.9–6.0] in plaques from individuals without diabetes vs. (p = 0.764, expressed as N/mm^2^, Fig. 4b).

## Discussion

In this study, we demonstrated that in individuals with T2DM with PAD and without MVD the percentage of CD14^+^CD16^++^ non-classical monocytes is decreased. The proportion of Tie2^+^ cells is increased within the CD14^++^CD16^+^ intermediate monocyte subpopulation, and this is accompanied by increased plasma levels of the Tie2 ligands Ang1 and Ang2. Since various exclusion criteria were applied (e.g. presence of microvascular disease), it is as yet unknown if the reported data apply to all individuals with T2DM. Intraplaque macrophage influx and angiogenesis did not differ in carotid plaques obtained from individuals with and without T2DM. 

Previous investigations into the association between monocyte subsets and cardiovascular disease have yielded inconclusive results. Monocyte heterogeneity may play a role in this, since monocytes are able to acquire inflammatory effector functions, but, in contrast, can also have protective effects in relation to tissue repair [41, 42]. The total pool of CD16^+^ monocytes has consistently been shown to be expanded in inflammatory diseases such as sepsis, rheumatoid arthritis, and infection [9, 43–45]. In addition, the total CD16^+^ monocyte population is increased in individuals with CAD [46]. However, results on the association of different CD16^+^ monocyte subsets and CVD are inconclusive. In individuals with chronic kidney disease, increased numbers of CD14^++^CD16^+^ intermediate monocytes were associated with higher cardiovascular mortality suggesting a detrimental role for these cells [17]. Moreover, this subset was shown to independently predict cardiovascular events in a cohort of individuals with suspected coronary artery disease referred for coronary angiography [18]. In contrast, high CD14^++^CD16^+^ intermediate monocyte levels were related to better outcomes following individuals with symptomatic ischemic heart failure [19]. The intermediate monocytes have been linked to a high cardiovascular risk profile [35] and reduced frequency of intermediate monocytes was linked to a reduction of subclinical atherosclerosis in morbidly obese individuals undergoing weight loss, including individuals with diabetes [47]. This suggests a positive correlation between circulating intermediate monocyte levels and the progression of atherosclerosis. However, levels of this monocyte subtype were inversely related to mortality in stroke individuals suggesting a protective role for these cells [20]. It was shown that the percentage of CD14^+^CD16^++^ non-classical monocytes was decreased in individuals with stable CAD and five or more cardiovascular risk factors, suggesting that this subset might be protective for CVD development [35]. Recently, it was demonstrated that the classical monocyte subset includes four subpopulations and within the non-classical monocyte subset also three subpopulations can be identified [48]. Investigation of these subsets might result in valuable information about monocytes and their relation with MVD, but this is beyond the scope of the current work. These results underscore the complexity of the potential role (protective vs. detrimental) of monocyte heterogeneity in atherogenesis, let alone in the presence of diabetes [49].

The proportion of CD16^+^ monocytes of the total CD14^+^ monocyte pool was found to be reduced in individuals with T2DM with vascular complications [50]. We did not observe a reduction in the percentage of total CD16^+^ monocytes in individuals with T2DM with MVD. However, in the abovementioned study, diabetes duration was longer than in our study (19.5 ± 1.1 vs. 14.0 ± 1.0 years respectively, p < 0.001). Moreover, individuals with both microvascular and macrovascular complications were compared to individuals with T2DM without complications, while in the present study, only individuals with T2DM with and without macrovascular complications were included. In addition, Fadini et al. reported no difference in the percentage of monocyte subsets between individuals with T2DM and healthy controls [51], whereas in our study, the percentage of non-classical monocytes was significantly reduced in individuals with T2DM. These clear differences in the study populations might have contributed to the different results obtained.

We subdivided the CD16^+^ monocyte population in CD14^++^CD16^+^ intermediate monocytes and CD14^+^CD16^++^ non-classical monocytes [13]. The percentage of CD14^+^CD16^++^ non-classical monocytes was decreased in individuals with T2DM with PAD or diabetic individuals without MVD, leading to a skewed ratio of non-classical/intermediate monocyte subsets in favour of CD14^++^CD16^+^ intermediate monocytes. Of note, this ratio was also significantly altered in individuals with T2DM without prevalent MVD, suggesting that diabetes itself rather than MVD affects CD16^+^ monocyte heterogeneity in these individuals. This shift is possibly caused by underlying changes in metabolism and the chronic inflammatory state in individuals with T2DM, which could affect monocyte phenotype [52]. In line with this, it was shown that hyperglycaemia shifts the differentiation of bone marrow-derived myeloid endothelial progenitor cells (EPCs) to a proinflammatory phenotype [53]. EPCs are a subset of monocytes known to be reduced in T2DM, as we demonstrated previously [36]. Also in a previous study in which samples from the Athero-Express biobank were analysed, the association between monocyte subsets and plaque vulnerability was studied [54]. Similar to current study, no differences in absolute numbers of different monocyte subsets were observed between individuals with diabetes and non-diabetic individuals. These data indicate that the observed differences in current study primarily originate from relative differences between subsets rather than absolute cell numbers. It has been suggested that monocyte heterogeneity represents a differentiation pathway from circulating classical to intermediate to non-classical monocytes, rather than the existence of discrete monocyte subsets [13, 15]. Therefore, a potential mechanism for the observed reduction in the percentage of non-classical monocytes in individuals with T2DM is the impairment of differentiation of intermediate monocytes into non-classical monocytes.

It has been shown that the presence of TEMs in tumour tissue is associated with tumour angiogenesis [23, 29]. Based on these data, as well as due to their proangiogenic phenotype and the potential detrimental role of plaque angiogenesis in the progression of atherosclerosis and plaque vulnerability [55], we hypothesized that proangiogenic TEMs are increased in individuals with T2DM. Our data indeed indicate that the frequency of Tie2^+^ cells within the CD14^++^CD16^+^ intermediate monocyte subset is markedly increased in individuals with T2DM with and without MVD compared to healthy controls. Only a few studies have been published in which TEMs have been studied in relation to CVD. In individuals with critical limb ischemia, TEM numbers were increased in the ischemic muscle in contrast to normoxic muscles from the same patient [56], which underlines the concept that hypoxia stimulates angiogenesis [3]. In line with this, HIF-1alpha expression, as marker of hypoxia, was detected in atherosclerotic plaques and was associated with VEGF expression and plaque inflammation [57]. In a pilot evaluation of venous blood in individuals with diabetes, TEMs were significantly increased in individuals with diabetes without ischemia in contrast to those with ischemia and healthy controls [33]. The authors of this study pointed out that they were less successful in extracting Tie2^+^ monocytes in individuals with diabetes and critical ischemia, which possibly affects the reliability of the results of the latter group. The study of Uçkay et al. is the only study describing TEMs in diabetes until now, in which it is suggested that neoangiogenesis, stimulated by TEMs, requires further investigation in those high-risk individuals. While limb ischemia is a result of PAD, in the current study, we did not find a selective increase of TEMs in individuals with T2DM and PAD but rather an increase in T2DM in general. In contrast to their study, in which TEMs were suggested to have protective effects, we propose that TEMs may act as a double-edged sword and contribute to accelerated atherosclerosis development in individuals with T2DM, potentially by aggravating intraplaque angiogenesis. 

The increase in CD14^++^CD16^+^Tie2^+^ monocytes in individuals with T2DM was accompanied by increased plasma levels of Tie2 ligands Ang1 and Ang2 in individuals with T2DM. Elevated levels of Ang2 in individuals with T2DM with and without CVD and in individuals with T2DM with retinopathy have been reported before [58, 59]. In line with this, also in our study Ang2 levels were overall increased in individuals with T2DM, however with a significant additional increase in individuals with T2DM and MVD. This additional increase might result from increased endothelial dysfunction and damage in individuals with MVD. Ang2 acts as a chemoattractant for Tie2^+^ monocytes and enhances the proangiogenic function of these cells [27, 30]. Therefore, exposure of already elevated numbers of Tie2^+^ monocytes in individuals with T2DM and MVD to these additionally increased levels of circulating Ang2 might further exacerbate the contribution of these cells to plaque instability and rupture. A potential limitation of the TEM analysis is that the staining could be performed on only a subset of the individuals because of availability. However, when looking at the patient characteristics of only the subjects used for the TEM analyses it appeared that these were comparable to the total cohort indicating that this was a representative subcohort.

In contrast to our hypothesis, an increased influx of macrophages and the number of intraplaque vessels was not found in endarterectomized carotid plaques obtained from individuals with T2DM compared to those without diabetes. Although it was previously shown that absolute numbers of circulating monocyte subsets were not associated with vulnerable plaque characteristics [54], a direct comparison of characteristics of plaques from individuals with or without T2DM could not be made in this study. Since we did not observe increased macrophage influx in the plaques from individuals with T2DM, it is unlikely that non-classical monocytes selectively migrated into plaques under diabetic conditions. The presence of a similar degree of macrophage influx and neovascularisation in current study might be explained by the stage of disease (i.e., degree of stenosis) of the included plaques. In this study, only advanced carotid plaques from individuals who underwent a surgical endarterectomy procedure were included, which is, for symptomatic or progressive asymptomatic atherosclerosis, indicated when diameter stenosis is 70–99% [39]. The monocyte subsets in general and differences between diabetic and non-diabetic circulating monocyte subsets possibly affect earlier stages and kinetics of arterial disease whereas endarterectomised plaques instead reflect an end-stage situation with a similar symptomatic plaque phenotype. Furthermore, in current study total macrophage influx was analysed using CD68 immunohistochemistry and no subdivision was made in different macrophage subsets. It was previously shown that pro-inflammatory M1 macrophages dominate the rupture-prone regions while vascular adventitial tissue shows an activated M2 profile. This suggests that macrophage-mediated inflammation is indeed associated with plaque vulnerability [60]. This dichotomous subdivision might be oversimplified since a recent follow-up study using single cell RNA sequencing identified five myeloid subsets in atherosclerotic plaques among which three different macrophage populations [61]. The exact role of these subsets in the pathogenesis of atherosclerosis remains to be elucidated. A post-mortem study investigated coronary lesions and demonstrated that lesions of individuals with T2DM included more intimal macrophages and T-cells [62]. This post-mortem study also found that the coronary plaque burden is increased in individuals with T2DM. Since we did not find differences in plaque surface area between plaques from diabetic and non-diabetic individuals, this underlines that the stage of arterial disease may play an important role in the intraplaque inflammation. Next to that, our immune phenotyping was performed in the blood of a non-corresponding, but similar high CVD risk cohort and did, therefore, not directly correspond to the included carotid plaques in this study.

## Conclusions

In conclusion, this study demonstrates that the percentage of non-classical monocytes is reduced and TEMs are increased in a substantial part of the individuals with T2DM. This suggests that the diabetic state alters monocyte heterogeneity, with potential implications for the development of MVD. Whether these monocytes stimulate atherogenesis in T2DM needs to be established.

## Data Availability

The datasets used and/or analysed during the current study are available from the corresponding author on reasonable request.

## Abbreviations

Ang1: Angiopoietin-1
Ang2: Angiopoietin-2
CAD: Coronary artery disease
CD: Cluster of differentiation
MVD: Macrovascular disease
PAD: Peripheral artery disease
T2DM: Type 2 diabetes mellitus
TEM: Tie2 expressing monocyte
Tie2: Tunica intima endothelial kinase 2
